# Genetic Diversity of Weedy Rice and Its Potential Application as a Novel Source of Disease Resistance

**DOI:** 10.3390/plants12152850

**Published:** 2023-08-02

**Authors:** Aron Osakina, Yulin Jia

**Affiliations:** 1 Department of Biology, Washington University in St. Louis, St. Louis, MO 63130, USA; aron.osakina@usda.gov; 2 USDA ARS Dale Bumpers National Rice Research Center, Stuttgart, AR 72160, USA

**Keywords:** disease resistance, rice blast disease, sheath blight disease, weedy rice

## Abstract

Weeds that infest crops are a primary factor limiting agricultural productivity worldwide. Weedy rice, also called red rice, has experienced independent evolutionary events through gene flow from wild rice relatives and de-domestication from cultivated rice. Each evolutionary event supplied/equipped weedy rice with competitive abilities that allowed it to thrive with cultivated rice and severely reduce yields in rice fields. Understanding how competitiveness evolves is important not only for noxious agricultural weed management but also for the transfer of weedy rice traits to cultivated rice. Molecular studies of weedy rice using simple sequence repeat (SSR), restriction fragment length polymorphism (RFLP), and whole-genome sequence have shown great genetic variations in weedy rice populations globally. These variations are evident both at the whole-genome and at the single-allele level, including *Sh4* (shattering), *Hd1* (heading and flowering), and *Rc* (pericarp pigmentation). The goal of this review is to describe the genetic diversity of current weedy rice germplasm and the significance of weedy rice germplasm as a novel source of disease resistance. Understanding these variations, especially at an allelic level, is also crucial as individual loci that control important traits can be of great target to rice breeders.

## 1. Introduction

**Figure 1:**
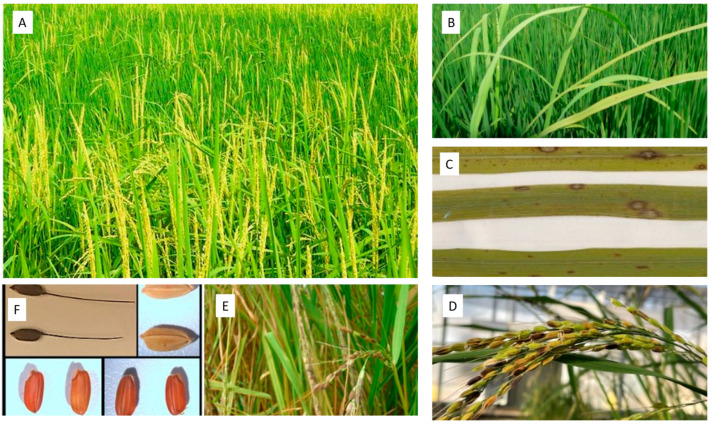
Weedy red rice in the USA. Weedy rice in a commercial rice field in Arkansas county (**A**). Blast reactions on leaves of weedy rice in a commercial Arkansas rice field (**B**) and enlarged leaves showing disease susceptibility (upper) and resistance (lower) (**C**). A black-hull awned (BHA) weedy rice genotype grown in a greenhouse at DB NRRC, Stuttgart, Arkansas (**D**), and in a rice field (**E**), and seeds of both BHA and straw-hull red rice from an Arkansas commercial rice field (**F**).

Rice (*Oryza sativa* L.) is one of the most important staple foods for humanity. Being an important food crop, research on agronomic traits aimed at improving rice production has been performed for domestication purposes. These traits include shattering, and genetic studies against shattering established *qSH1* and *Sh4* through QTL mapping to be key alleles for reduced shattering in cultivated rice; they have been well studied for domestication in cultivated rice [1,2,3]. Another important trait selected for domestication in cultivated rice is the pericarp color pigmentation, which is controlled by the *Rc* allele that encodes a basic helix–loop–helix (bHLH) protein and results in a grain color change from red to white [4]. Moreover, the domestication process in cultivated rice was changed in flowering time; variations in flowering times allowed farmers to broaden their growing areas [5,6]. The *Hd1* involved in the regulation of photoperiodic flowering [7,8,9,10] was shown to be the major determiner of flowering time in cultivated rice [9]. Although to date no clear investigation has been reported on cultivated rice to conclude the selection of *Hd1* locus for domestication, mutations in *Hd1* resulting in variation in flowering time previously reported by Takahashi et al. [9] indicate that it could be a potential candidate for domestication selection and requires further detailed investigation. Weedy rice is one of the most troublesome agricultural issues globally because it competes for nutrients, water, sunlight, and other crucial vital resources with cultivated rice, thus posing a great threat to food security [11,12]. Owing to its early flowering, enhanced photosynthesis, and rapid grain filling, weedy rice can successfully outcompete cultivated rice [13,14,15,16]. Weedy rice is morphologically similar to cultivated rice during early stages of growth but possesses undesirable traits that distinguish it from cultivated rice; these traits, such as heavy shattering that allows dispersal of seeds [17,18] and red pericarp pigmentation [19,20,21], when present in white-pericarp cultivated rice tend to lower the grain quality and seed dormancy that enables continued existence of weedy rice in cultivated rice fields [22]. Another advantage weedy rice has over cultivated rice that is that it has been shown to exhibit photoperiod sensitivity and flowering time variation, and increased plant height when compared to cultivated rice [7,8,9,10]. Moreover, some weedy rice biotypes possess awns that offer protection from predation. [21]. Each year, weedy rice causes significant economic loss in rice production due to reduced quantity and grain quality [23,24]. Selective elimination of weedy rice from cultivated rice fields through application of herbicide has had limited success, since weedy and cultivated rice share morphological and physiological traits [25]. Understanding the biology of weedy rice is essential for weed management and can also benefit crop protection. Molecular markers such as microsatellite or simple sequence repeats (SSR), amplified fragment length polymorphism (AFLPs), random amplified polymorphism DNA (RAPDs), and DNA sequencing have been developed and applied in the study of genetic variations and conservation studies by biologists in different plant species [26,27]. For instance, microsatellite markers have been used in conservation studies in endangered plant species such as *Calystegia soldanella* [28] and *Tricyrtis ishiiana* [29]. AFLP has been applied in genetic variations studies in plants such as *Jatropha curcas* [30] and *Rhodiola rosea* [31]. SSR is the most used molecular marker and is regularly deployed as allele-specific and co-dominant markers in assessing the genetic diversity and evolutionary relationships in wild and weedy rice [32,33]. In weedy rice, apart from SSR markers, genome sequencing and quantitative trait loci analysis (QTL) have also been used to explore the genetic diversity [34,35]. In this short review, we describe the genetic diversity at the allelic and genomic level in weedy rice and propose weedy rice as a novel source for disease resistance in cultivated rice. Morphological characteristics of weedy rice population obtained in rice commercial rice fields in Arkansas (Figure 1).

## 2. Diversity at an Allelic Level

### 2.1. Seed-Shattering Gene (Sh4)

Seed shattering allows plants to shed their seeds from the plant to the field and is crucial for dispersal [36]. Seed shattering is costly to farmers, as it leads to crop yield reduction and may cause continued presence of volunteer plants in cultivated fields [37,38]. Shattering of seeds by wild or weedy plants is considered a fitness mechanism that allows plants to evade collection by farmers for destruction. At maturity, shattering is sometimes crucial as it allows seeds to retain sufficient moisture for dormancy, thus enabling weeds to survive during winter and germinate during the cropping season [22,39,40]. Seed shattering is controlled by a complex genetic mechanism and involves proper formation and subsequent degradation of an abscission layer [41]. Quantitative trait loci (QTLs) involved in seed shattering have been identified on almost all rice chromosomes [3,42,43,44,45,46]. To date, major genes involved in rice seed shattering have been cloned. These genes include *qSH1*, located on chromosome 1, which encodes a BEL1-type homeobox. A single nucleotide polymorphism (SNP) that is present at the 5′ upstream regulatory region of *qSH1* represses *qSH1* expression from the abscission layer, causing reduced shattering in cultivated rice [2]. *Sh*-*h*, which encodes a C-terminal domain phosphatase-like protein located on chromosome 7, inhibits the development of abscission zones in cultivated rice; inactivation of *Sh*-*h* caused increased shattering in mutant strains, confirming *Sh*-*h* is a negative regulator of shattering in rice [47]. *SHATI*, which encodes APETALA2 transcription factor, located in chromosome 4, was demonstrated to be important for shattering by specifying the abscission zone formation [41], while *Sh4*, a member of the trihelix family of transcription factors, controls shattering via hydrolyzing AZ cells during the abscission process [1,48]. Among the four previously cloned genes, *Sh4* is located on chromosome 4 and is most responsible for reduced seed shattering in rice [48]. This gene was isolated from progeny derived from a cross between cultivated *O. sativa* subpopulation *indica* and a wild seed-shattering species of *O. nivara*. The *Sh4* gene was shown to be involved in the degradation of the abscission layer between the grain and the pedicel [48]. A nonsynonymous nucleotide substitution from G to T is present in the first exon of *Sh4* of cultivated rice. This substitution was reported to be the main reason for reduced *Sh4* function [48]. Functional genomic studies showed that when *Sh4* was edited in weedy rice, seed shattering was reduced [49]. Many cultivated rice varieties have been shown to develop shattering due to reversion to the wild rice populations through de-domestication (endoferality) [37,38]. Reduced shattering in cultivated rice has been linked to a T mutation in exon one of the *Sh4* gene; however, several studies on weedy rice have shown that the T mutation is not the sole reason for observed reduced shattering. Thurber et al. reported that despite possessing a T mutation at the *Sh4* locus like cultivated rice, most US weedy rice displays high shattering ability. The presence of non-shattering T mutations in US weedy rice as reported by Thurber et al. [50] suggests they can be a major unidentified locus or several minor loci responsible for shattering within *Oryza* that are hard to detect. Similarly, 20 out of 24 Italian weedy accessions had a G-to-T mutation at *Sh4* locus and displayed high shattering ability [19]. The non-shattering *Sh4* genotype was also reported in Asian weedy rice but showed a shattering phenotype [18]. Other genetic studies showed that the G-to-T mutations at the *Sh* locus caused reduced seed shattering in weedy rice [51,52]. Some diversity studies at the *Sh4* locus have shown weedy rice to contain both G and T and heterozygous GT nucleotides. For example, Song et al. [51] reported that 178 accessions of Malaysia weedy rice displayed increased shattering and comprised accessions with fixed reduced shattering T and shattering G alleles. Of 178 accessions, 104 (58.4%) were shown to be T homozygotes, 63 (35.4%) were homozygous for the ancestral G allele, and the remaining 11 (6.2%) displayed G/T heterozygosity. Similarly, phylogenetic analysis of South Asia weedy rice has shown large variations among the *Sh* alleles. This South Asia weedy rice was classified as wild-like, and all had the ancestral G allele. The remaining *aus*- and *indica*-type weedy rice had the T allele except only two [52]. The T allele was the majority according to Huang et al. [52] with a few having the G allele, thus suggesting the majority of Asian weedy rice originated from cultivated *aus* and *indica* varieties through de-domestication. Furthermore, variation studies at the *Sh4* allele on Thai weedy rice revealed the existence of a dominant T allele in 95 of 111 accessions (85.6%). This T allele was present in all cultivated rice (59 of 59 accessions). Of the remaining 16 (14.4%) weedy rice accessions, one was heterozygous (G/T), and 15 were homozygous (G/G) [53], indicating that Thai weedy rice had multiple independent origins of *Sh4*.

### 2.2. Diversity at the Hd1 Locus

The *Hd1* locus has been reported in many studies as being crucial for photoperiod sensitivity and flowering time variation in cultivated rice [7,8,9,10]. *Hd1* has been shown to promote flowering under short-day (SD) conditions, while long-day (LD) flowering is repressed [54,55]. Most cultivated rice contains either *SNPs* or deletions in the *Hd1* locus that render it nonfunctional, thus abolishing day-length sensitivity and resulting in later flowering under short days. A 2 bp deletion found in the exon is the most common mutation, and it causes premature stop codons and is present in some *indica* and some *japonica* cultivars [9]. Between the two US weedy rice genotypes, the SH weedy rice was reported to take less time to flower than the local tropical *japonica* cultivars, while the BHA weedy rice flowered at the same time or took longer than the cultivar [56,57]. The drastic difference in flowering exhibited by the SH and BHA was due to the *Hd1* alleles. The BHA weedy rice genotype has nonfunctional *Hd1* alleles, which lead to loss of day-length sensitivity and later flowering under short-day conditions, while most of the SH weeds carry functional *Hd1* alleles and are day-length-sensitive, resulting in early flowering under short-day conditions. Phenotypic traits such as height, emergence growth rate, average growth, flowering time, and tiller number grouped US weedy rice into four different haplotypes, SH, BHA (1 and 2), and BRH, with their progenitors being *indica*, *aus*, and hybridization of SH and BHA weeds (BRH), respectively [58]. The BHA (1 and 2) plants were taller (BHA1-85 CM, BHA2-99 CM) and took a long time to flower (BHA1 126 days, BHA2 111 days). Genetic diversity studies at the *Hd1* locus by Reagon et al. [58] showed that the BHA genotypes are highly diverse compared to other weedy rice genotypes. 

### 2.3. Diversity at the Rc Locus

Red pericarp is a prominent feature that distinguishes weedy rice from cultivated rice [20], and it is due to the buildup of anthocyanins and proanthocyanidins in the pericarp [19]. Accumulation of anthocyanins and proanthocyanidins in the pericarp is a crucial physiological function for promoting seed dormancy [59]. Two loci (*Rc* and *Rd*) that are responsible for red pericarp pigmentations in rice have been identified. These loci complement each other in their function. The *Rc* locus is responsible for the accumulation of pigments, while the *Rd* gene increases the content of the pigment in pericarp [60]. For red pericarp pigmentations to occur, both the *Rc* and *Rd* loci must be present. *Rc* alone produces brown seeds, while *Rd* without *Rc* has a white-pericarp phenotype [4]. The *Rc* gene is located on rice chromosome 7, contains eight exons, and encodes a basic helix–loop–helix transcription factor. This is an important gene for the regulation of proanthocyanidin biosynthesis [19,59]. Proanthocyanin accumulation results in red pericarp pigmentation and was shown to be dependent on functionality of the *Rc* allele [19]. Analysis of the *Rc* allele identified a 14 bp deletion in the seventh exon; this deletion is present in most white-pericarp rice cultivars. The deletion causes a frameshift that results in a premature stop codon, which inactivates the DNA-binding domain, thus making the transcription factor nonfunctional [61]. Moreover, Sweeney et al. [4] reported *Rc*-s to be present in other white-pericarp rice genotypes and is characterized by a C-to-A base transversion in the seventh exon. The alleles *Rc* and *Rc-s* result in an inactive transcription factor, causing the pericarp to be white [4]. Some studies have reported the existence of red pericarp in rice cultivars, and this phenotype is attributed by mutational reversion of the *Rc* nonfunctional allele to functional form. For instance, Brooks et al. [62] showed that 1 bp deletion is present at 20 bp upstream of the origin 14 bp deletion in the *Rc* allele; this mutation restores reading from protein function and the proanthocyanidin pigmentation in the US red-pericarp cultivar ‘Wells’. Similarly, the Italian cultivar ‘Perla’ has red pericarp pigmentation as reported by Lee et al. [63]. The red-pericarp phenotype is due to the presence of 1 bp deletion located at the 44 bp upstream of the 14 bp deletion in the *Rc* gene. These two studies [62,63] therefore indicate not all rice cultivars contain the nonfunctional *Rc* allele that has been selected for domestication. 

Weedy rice, also called red rice, is found in many rice-growing regions in the world. Previous genetic studies reported that red pericarp pigmentation is caused by a functional *Rc* gene in weedy rice resulting in the upregulation of proteins in the proanthocyanin biosynthetic pathway [19,20]. Although pericarp pigmentation is associated with a 14 bp deletion at the *Rc* allele resulting in loss of function, in other studies, Italian weedy rice was classified into two haplotypes based on pericarp pigmentation. Haplotype 1 accessions had red pericarp without the 14 bp deletion at the *Rc* locus, similar to the *Rc* allele in *O. rufipogon*, suggesting these haplotypes could have originated from the wild rice *O. rufipogon* population [19]. Haplotype 2 weedy rice accessions had a white pericarp with their *Rc* allele having the 14 bp deletion, which was similar to that of the japonica allele and an additional 1 bp deletion in the upstream region of the 14 bp gap. Thus, haplotype 2 might have evolved from japonica rice cultivars. Moreover, a diversity study at the *Rc* locus in Malaysia weedy rice showed that 43 out of 52 had a red pericarp, while the remaining 9 had white pericarps [64]. Direct Sanger sequencing of the exon 7 region at the *Rc* locus confirmed the presence of the 14 bp deletion in eight of the nine white-pericarp Malaysian weedy rice. The phylogenetic tree [64] categorized this Malaysian weedy rice into three distinct groups. The largest group of Malaysian weeds (32 of 52 accessions) is in a large clade (labeled group 1); they were genetically closer to United States weeds, red-pericarp domesticated rice, and a few *O. rufipogon* accessions. The second clade (group 2) contains seven Malaysian weeds that are grouped exclusively with *O. rufipogon* accessions, while the third group of Malaysian weeds (group 3) according to Cui et al. [64] is characterized by haplotypes that either carry the *Rc* 14 bp deletion or have functional *Rc* sequences closely related to *Rc* genotype. 

## 3. Diversity at the Genome Level

**Figure 2:**
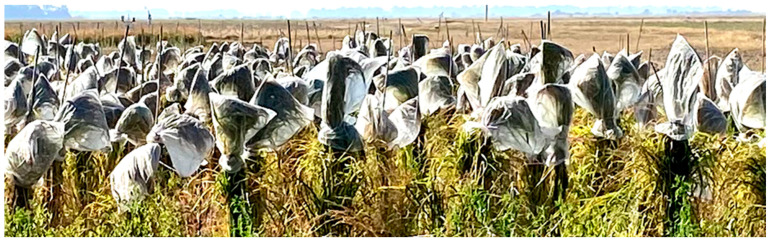
Weedy rice mapping population derived from a cross of US BHA genotype with aus growing in a rice field at Dale Bumpers National Rice Research Center in Stuttgart, Arkansas. Plants were bagged for seed collection.

Molecular markers and whole-genome sequencing have been used to study genetic variations in weedy rice. For example, Lu et al. [65] used InDel molecular markers and deployed principal component analysis to examine weedy rice in Asia. The study identified two different genetic weedy rice groups, the *indica* weedy rice accessions and the japonica weedy rice accessions. The *indica* varieties were found across latitudes between 5 to 40° N, while the *Japonica* types were mostly confined to latitudes > 35° [65]. Further analysis of the Asian weedy rice and common cultivated and wild rice by using Nei’s genetic distance showed that *japonica* Asian weedy rice genotypes were genetically closer to the local *japonica* cultivars, suggesting that these weedy rice accessions originating from *japonica* cultivars [65]. The *indica* weedy varieties are genetically closer to the common wild rice, suggesting that they could have gradually originated from common wild rice or from natural cross hybrids of *indica* cultivars and common wild rice [65]. Analysis of genetic diversity and origin of North Asia weedy rice with SSR markers indicated that weedy rice in this region was highly diverse genetically, with a heterozygosity of 0.434 and a high Shannon’s information index of 0.748 [66]. Moreover, the use of SSR markers with the application of cluster analysis (UPGMA) and (PCA) principal component analysis to analyze genetic diversity of 30 weedy rice in Liaoning province in China by Cao et al. [67] showed a relatively high diversity in the weedy population (He = 0.313, I = 0.572). Furthermore, molecular studies using SSRs identified two major genotypes of weedy rice in the USA. These were black hull with awn (BHA) with an estimated diversity of 0.76 and the straw-hull awnless (SH) with a diversity of 0.68, and they were shown to have originated from cultivated Asian *aus* and *indica* progenitors, respectively [57], of these two US genotypes, which was confirmed using additional microsatellite and single-nucleotide polymorphism (SNP) molecular analysis [17,68]. SSR markers have also been used to study the genetic diversity of Italian weedy rice, where the data of 19 SSR markers revealed that Italian weedy rice is highly diverse, with an allelic average and heterozygosity of 3.368 and 0.295, respectively [69]. Weedy rice from Uruguay was classified into three distinct groups, A, B, and C, using AFLP molecular markers. Genetic diversity of these clusters was verified using dendrograms where A and C were purely weedy type, while cluster B included cultivated rice varieties [70]. Genome sequences were used to study the genetic diversity in weedy rice. The whole-genome sequences of 183 wild, cultivated, and weedy rice accessions were analyzed to assess the origins of weedy rice genotypes in the USA [71]. The origins of the US BHA weedy rice diverging from its crop ancestor *aus* were found to be much earlier than SH and Chinese weedy rice, whose ancestor is *indica*. Only a few genomic changes were identified that could lead to the formation of weedy forms. Distinct genomes of the SH and BHA weedy rice may have resulted from parallel evolution. Some genomic regions showing footprints of selection overlapped with other weedy genetic loci, suggesting that parallel evolution has redefined the weedy rice genome. A mapping population derived from a cross of a BHA genotype with an aus was developed to evaluate the genetic basis of the competitiveness of weedy rice (Figure 2).

Whole-genome sequencing analysis of 30 Korean weedy rice genotypes showed that Korean weedy rice did not originate from wild rice relatives since they were distinct and arose from either japonica or *indica* cultivated rice [72].

## 4. Novel Source of Disease Resistance

**Figure 3:**
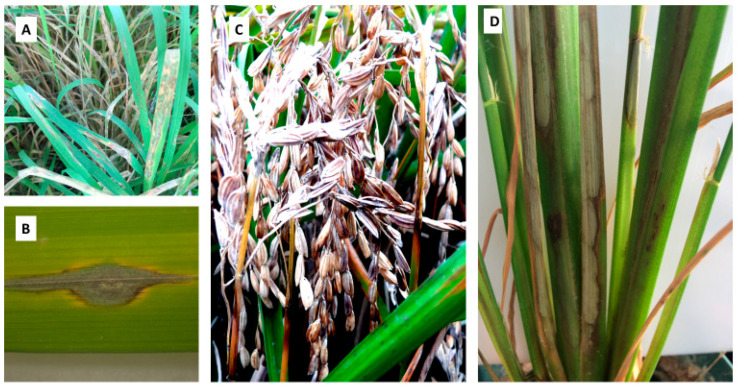
Graphic description of rice blast caused by *M. oryzae* and sheath blight disease caused by *R. solani* of cultivated rice. Seedling blast disease (**A**) enlarged typical diamond shape lesion of leaf blast (**B**), panicle blast showing 90% crop loss (**C**), and sheath blight disease of rice showing typical symptom on sheath (**D**).

Rice blast disease caused by the filamentous fungus *Magnaporthe oryzae* and sheath blight disease caused by the fungus *Rhizoctonia solani* are two major diseases threatening rice production worldwide (Figure 3) [73]. 

These two diseases have existed in commercial rice fields for hundreds of years, and weedy rice has adapted and evolved to survive these two biological stressors. Resistance to *M. oryzae* is governed by race-specific major resistance (*R*) genes and minor QTLs. Resistance to *R. solani* could be governed by QTLs. However, major *R* genes to *R. solani* have not been discovered in rice germplasm. Weedy rice, which competes with cultivated rice, possesses ancient untapped and novel *R* genes. For instance, Zhao et al. [74] studied the blast-resistant *Ptr* allele in black-hull weedy rice. The *Ptr* gene, previously named as *Pi-ta2* in rice, encodes a protein with four armadillo repeats conferring a broad spectrum of resistance except for blast race IB33 [75]. Race IB33 is one of the most virulent blast races identified on the plant in our laboratory but not found in commercial fields. Sequence analysis of the *Ptr* allele from weedy rice, PtrBHA, identified a unique amino acid, glutamine (Gln), at protein position 874. This amino acid is absent in susceptible individuals. Minor changes in protein conformation of PtrBHA are predicted to create novel resistance to race IB33 [74]. Using genotyping by sequencing (GBS), a total of 28 resistance QTLs were identified in two US weedy rice ecotypes [76]. These resistance QTLs, some with large effects and others with small effects, suggest that US weedy rice has adapted to blast disease using both major *R* genes and QTLs. These *R* genes have not been found in cultivated rice varieties, suggesting that they are newly evolved *R* genes. In another study, sheath blight resistance QTLs were identified using two recombinant inbred line mapping populations derived from crosses of an *indica* crop variety, Dee-Geo-Woo-Gen (DGWG), with progeny representing straw-hull (SH) and black-hull awned (BHA) [77]. A total of nine QTLs were identified, five of which were attributable to alleles for plant height and days to heading. Four sheath blight resistance QTLs were identified by treating these growth traits as covariates. Two of these QTLs, *qShB1-2* and *qShB4*, are new and were not identified in the previous study by Jia et al. [78]. *Pi*-*ta* is another effective *R* gene deployed to control rice blast disease in many rice-growing regions of the world. Weedy rice genotypes containing the resistant *Pi*-*ta* allele showed a high level of resistance to two predominant US blast races, IB49 and IC17 rice [79]. The *Pi*-*ta* gene on rice chromosome 12 encodes a predicted nucleotide binding site and leucine-rich domain which directly interacts with the product of the *M. oryzae* avirulence gene *AVR-Pita1* during resistant responses [80]. The genome organization of the *Pi*-*ta* gene in weedy rice was investigated in a few studies to determine if gene flow between cultivated and weedy rice had occurred in the USA [81]. The resistant *Pi*-*ta* allele was found in most of the investigated US weedy rice genotypes. The genomic region with the *Pi*-*ta* allele in US weedy rice was found to be very similar to that of cultivated rice [80]. The flanking sequences of the *Pi*-*ta* gene and SSR marker analysis revealed that the susceptible *Pi*-*ta* allele and the non-resistant *Pi*-*ta* allele had been introgressed from US cultivated rice to weedy rice through gene flow. This may be because the *Pi*-*ta* gene has not been widely deployed in the USA. In conclusion, these findings on rice blast and sheath blast diseases demonstrate that novel *R* genes from weedy rice can be used in combination with favorable growth traits to develop rice germplasms that are resistant to rice blast and sheath blast. 

## 5. Conclusions and Prospects

Weedy rice is one of the most damaging weeds for rice production. In this review, we summarized research on seed shattering, photoperiod sensitivity, flowering, and resistance to two major diseases. We have described that increased seed shattering in cultivated rice is controlled by several QTLs [3,42,43,44,45,46], with *Sh4* being the most important for shattering. We have further described that reducing shattering in cultivated rice is due to the presence of G-to-T nonsynonymous mutation in the *Sh4* allele; however, this mutation is present in the majority of weedy rice that exhibited increased shattering, thus indicating the existence of unknown QTLs responsible for shattering in weedy rice, a promising subject for future studies.

Proanthocyanidins have been reported to contain numerous health benefits such as antioxidant, anticancer, antidiabetic, neuroprotective, and antimicrobial properties [82]. Furthermore, consumption of proanthocyanidins containing food products, such as red wine and chocolate, appeared to be associated with lower blood pressure, insulin resistance, and reverse endothelial dysfunction [83,84,85]. Weedy red rice genotypes have been reported to contain high levels of proanthocyanidin. An attempt to increase proanthocyanidins content in red rice through crossing red-pericarp ‘Hong Xiang 1’ (‘HX1’) and white-pericarp rice ‘Song 98-131’ (‘S98-131’) was undertaken [86]; however, this approach is not applicable for crossing weedy rice and cultivated rice as this could result in introgression of undesirable weedy traits in rice cultivars. On the other hand, with extensive genetic studies on weedy rice, alleles controlling pericarp pigmentation can individually be exploited. Weedy rice genotypes without the 14 bp deletion at the *Rc* functional locus have been reported to contain high levels of proanthocyanidins in their pericarp [19]; their respective *Rc* locus can be introduced in rice varieties with low proanthocyanidins through gene editing to generate rice varieties with higher or improved proanthocyanidins quantities.

Reduced flowering time is crucial as it tends to reduce the production period. The flowering time is controlled by *Hd1*, which activates flowering during short days [54,55]. Although weedy rice infestation is a serious threat to rice production qualitatively and quantitatively, the *Hd1* locus especially of US SH weedy rice genotypes that were reported to flower earlier than cultivated rice [57,58] can individually be introgressed in cultivated rice via gene editing to develop commercial rice cultivars with a short flowering time, thus reducing time periods required for seed production.

Plant diseases are the major causes of substantial yield losses each year and are of great threat to food security and agricultural sustainability globally. Managing plant diseases through creating resistant crop germplasm is therefore a noble practice geared towards minimizing crop losses. Rice production continues to be threatened by rice blast and sheath blast [83], but the US black-hull awned (BHA) weedy rice genotype has been shown to be resistant to these diseases. The resistance spectra to blast disease by US black-hull awned (BHA) weedy rice was attributed to the *Ptr* and *Pi*-*ta* genes [75,79], while resistance to sheath blight was due to mapped QTLs [77,78]. This black-hull awned (BHA) weedy rice is therefore an ideal candidate for further conclusive screening for the identification of disease resistance genes (our current ongoing study). The resistance gene to be established and the already reported ones can then be edited in cultivated rice to generate resistant varieties. In conclusion, this review highlights the genetic diversity of the present weedy rice germplasm, confirming that weedy rice has multiple independent origins and that beneficial alleles contained in weedy rice germplasm can be tapped in breeding experiments to generate resistant rice varieties with beneficial nutritional value using gene-editing techniques.

## Data Availability

Not applicable.

## References

[1] Li C., Zhou A., Sang T. (2006). Rice Domestication by Reducing Shattering. Science.

[2] Konishi S., Izawa T., Lin S.Y., Ebana K., Fukuta Y., Sasaki T., Yano M. (2006). An SNP Caused Loss of Seed Shattering During Rice Domestication. Science.

[3] Zhang L.B., Zhu Q., Wu Z.Q., Ross-Ibarra J., Gaut B.S., Ge S., Sang T. (2009). Selection on Grain Shattering Genes and Rates of Rice Domestication. New Phytol..

[4] Sweeney M.T., Thomson M.J., Pfeil B.E., McCouch S. (2006). Caught Red-Handed: *Rc* Encodes a Basic Helix-Loop-Helix Protein Conditioning Red Pericarp in Rice. Plant Cell.

[5] Jung C., Müller A.E. (2009). Flowering Time Control and Applications in Plant Breeding. Trends Plant Sci..

[6] Izawa T. (2007). Adaptation of Flowering-Time by Natural and Artificial Selection in Arabidopsis and Rice. J. Exp. Bot..

[7] Fujino K., Wu J., Sekiguchi H., Ito T., Izawa T., Matsumoto T. (2010). Multiple Introgression Events Surrounding the *Hd1* Flowering-Time Gene in Cultivated Rice, *Oryza sativa* L.. Mol. Genet. Genom..

[8] Subudhi P.K., De Leon T.B., Tapia R., Chai C., Karan R., Ontoy J., Singh P.K. (2018). Genetic Interaction Involving Photoperiod-Responsive *Hd1* Promotes Early Flowering under Long-Day Conditions in Rice. Sci. Rep..

[9] Takahashi Y., Teshima K.M., Yokoi S., Innan H., Shimamoto K. (2009). Variations in *Hd1* Proteins, *Hd3a* Promoters, and *Ehd1* Expression Levels Contribute to Diversity of Flowering Time in Cultivated Rice. Proc. Natl. Acad. Sci. USA.

[10] Yano M., Katayose Y., Ashikari M., Yamanouchi U., Monna L., Fuse T., Baba T., Yamamoto K., Umehara Y., Nagamura Y. (2000). *Hd1*, a Major Photoperiod Sensitivity Quantitative Trait Locus in Rice, Is Closely Related to the Arabidopsis Flowering Time Gene CONSTANS. Plant Cell.

[11] Estorninos L.E., Gealy D.R., Gbur E.E., Talbert R.E., Mcclelland M.R. (2005). Rice and Red Rice Interference. II. Rice Response to Population Densities of Three Red Rice (*Oryza sativa*) Ecotypes. Weedy Sci..

[12] Dekker J. (1997). Weed Diversity and Weed Management. In Proceedings of the Weed Science. Weed Sci. Soc. Am..

[13] Burgos N.R., Norman R.J., Gealy D.R., Black H. (2006). Competitive N Uptake between Rice and Weedy Rice. Field Crops Res..

[14] Dai L., Dai W., Song X., Lu B., Qiang S. (2014). A Comparative Study of Competitiveness between Different Genotypes of Weedy Rice (*Oryza sativa*) and Cultivated Rice. Pest Manag. Sci..

[15] Dai L., Song X., He B., Valverde B.E., Qiang S. (2017). Enhanced Photosynthesis Endows Seedling Growth Vigour Contributing to the Competitive Dominance of Weedy Rice over Cultivated Rice. Pest Manag. Sci..

[16] Zhao C., Xu W., Song X., Dai W., Dai L., Zhang Z., Qiang S. (2018). Early Flowering and Rapid Grain Filling Determine Early Maturity and Escape from Harvesting in Weedy Rice. Pest Manag. Sci..

[17] Reagon M., Thurber C.S., Gross B.L., Olsen K.M., Jia Y., Caicedo A.L. (2010). Genomic Patterns of Nucleotide Diversity in Divergent Populations of U.S. Weedy Rice. BMC Evol. Biol..

[18] Zhu Y., Ellstrand N.C., Lu B.R. (2012). Sequence Polymorphisms in Wild, Weedy, and Cultivated Rice Suggest Seed-Shattering Locus *Sh4* Played a Minor Role in Asian Rice Domestication. Ecol. Evol..

[19] Grimm A., Sahi V.P., Amann M., Vidotto F., Fogliatto S., Devos K.M., Ferrero A., Nick P. (2020). Italian Weedy Rice—A Case of de-Domestication?. Ecol. Evol..

[20] Gross B.L., Reagon M., Hsu S.C., Caicedo A.L., Jia Y., Olsen K.M. (2010). Seeing Red: The Origin of Grain Pigmentation in US Weedy Rice. Mol. Ecol..

[21] Delouche J.C. (2007). Diversity of Weedy Rice Populations Weedy Rices-Origin, Biology, Ecology and Control.

[22] Gu X.Y., Kianian S.F., Foley M.E. (2004). Multiple Loci and Epistases Control Genetic Variation for Seed Dormancy in Weedy Rice (*Oryza sativa*). Genetics.

[23] Eleftherohorinos I.G., Dhima K.V., Vasilakoglou I.B. (2002). Interference of Red Rice in Rice Grown in Greece. Weed Sci..

[24] Ziska L.H., Gealy D.R., Burgos N., Caicedo A.L., Gressel J., Lawton-Rauh A.L., Avila L.A., Theisen G., Norsworthy J., Ferrero A. (2015). Weedy (Red) Rice. An Emerging Constraint to Global Rice Production. Adv. Agron..

[25] Olofsdotter M., Valverde B.E., Madsen K.H. (2000). Herbicide Resistant Rice (*Oryza sativa* L.)*:* Global Implications for Weedy Rice and Weed Management. Ann. Appl. Biol..

[26] Arif I.A., Bakir M.A., Khan H.A., Al Farhan A.H., Al Homaidan A.A., Bahkali A.H., Al Sadoon M., Shobrak M. (2010). A Brief Review of Molecular Techniques to Assess Plant Diversity. Int. J. Mol. Sci..

[27] O’Hanlon P.C., Peakall R., Briese D.T. (2000). A Review of New PCR-Based Genetic Markers and Their Utility to Weed Ecology. Weed Res..

[28] Noda A., Nomura N., Mitsui Y., Setoguchi H. (2009). Isolation and Characterisation of Microsatellite Loci in *Calystegia soldanella* (Convolvulaceae), an Endangered Coastal Plant Isolated in Lake Biwa, Japan. Conserv. Genet..

[29] Setoguchi H., Mitsui Y., Ikeda H., Nomura N., Tamura A. (2009). Development and Characterization of Microsatellite Loci in the Endangered *Tricyrtis ishiiana* (Convallariaceae), a Local Endemic Plant in Japan. Conserv. Genet..

[30] Shen J., Pinyopusarerk K., Bush D., Chen X. (2012). AFLP-Based Molecular Characterization of 63 Populations of *Jatropha curcas* L. Grown in Provenance Trials in China and Vietnam. Biomass Bioenergy.

[31] Elameen A., Klemsdal S.S., Dragland S., Fjellheim S., Rognli O.A. (2008). Genetic Diversity in a Germplasm Collection of Roseroot (*Rhodiola rosea*) in Norway Studied by AFLP. Biochem. Syst. Ecol..

[32] Song Z.P., Xu X., Wang B., Chen J.K., Lu B.R. (2003). Genetic Diversity in the Northernmost *Oryza rufipogon* Populations Estimated by SSR Markers. Theor. Appl. Genet..

[33] Yu G.Q., Bao Y., Shi C.H., Dong C.Q., Ge S. (2005). Genetic Diversity and Population Differentiation of Liaoning Weedy Rice Detected by RAPD and SSR Markers. Biochem. Genet..

[34] Yao N., Wang L., Yan H., Liu Y., Lu B.R. (2015). Mapping Quantitative Trait Loci (QTL) Determining Seed-Shattering in Weedy Rice: Evolution of Seed Shattering in Weedy Rice through de-Domestication. Euphytica.

[35] Qiu J., Zhu J., Fu F., Ye C.Y., Wang W., Mao L., Lin Z., Chen L., Zhang H., Guo L. (2014). Genome Re-Sequencing Suggested a Weedy Rice Origin from Domesticated *Indica-Japonica* Hybridization: A Case Study from Southern China. Planta.

[36] Dong Y., Wang Y.Z. (2015). Seed Shattering: From Models to Crops. Front. Plant Sci..

[37] Vigueira C.C., Li W., Olsen K.M. (2013). The Role of *Bh4* in Parallel Evolution of Hull Colour in Domesticated and Weedy Rice. J. Evol. Biol..

[38] Roberts J.A., Whitelaw C.A., Gonzalez-Carranza Z.H., McManus M.T. (2000). Cell Separation Processes in Plants-Models, Mechanisms and Manipulation. Ann. Bot..

[39] Gu X.Y., Kianian S.F., Foley M.E. (2005). Seed Dormancy Imposed by Covering Tissues Interrelates to Shattering and Seed Morphological Characteristics in Weedy Rice. Crop Sci..

[40] Gu X.Y., Kianian S.F., Hareland G.A., Hoffer B.L., Foley M.E. (2005). Genetic Analysis of Adaptive Syndromes Interrelated with Seed Dormancy in Weedy Rice (*Oryza sativa*). Theor. Appl. Genet..

[41] Zhou Y., Lu D., Li C., Luo J., Zhu B.F., Zhu J., Shangguan Y., Wang Z., Sang T., Zhou B. (2012). Genetic Control of Seed Shattering in Rice by the *APETALA2* Transcription Factor Shattering Abortion1. Plant Cell.

[42] Lee G.H., Kang I.K., Kim K.M. (2016). Mapping of Novel QTL Regulating Grain Shattering Using Doubled Haploid Population in Rice (*Oryza sativa* L.). Int. J. Genom..

[43] Htun T.M., Inoue C., Chhourn O., Ishii T., Ishikawa R. (2014). Effect of Quantitative Trait Loci for Seed Shattering on Abscission Layer Formation in Asian Wild Rice *Oryza rufipogon*. Breed Sci..

[44] Xiong L.Z., Liu K.D., Dai X.K., Xu C.G., Zhang Q., Xu C.G., Zhang Q. (1999). Identification of genetic factors controlling domestication-related traits of rice using an F2 population of a cross between *Oryza sativa* and *O. rufipogon*. Theor. Appl. Genet..

[45] Tsujimura Y., Sugiyama S., Otsuka K., Htun T.M., Numaguchi K., Castillo C., Akagi T., Ishii T., Ishikawa R. (2019). Detection of a Novel Locus Involved in Non-Seed-Shattering Behaviour of *Japonica* Rice Cultivar, *Oryza sativa* ‘Nipponbare’. Theor. Appl. Genet..

[46] Subudhi P.K., Singh P.K., Deleon T., Parco A., Karan R., Biradar H., Cohn M.A., Sasaki T. (2014). Mapping of Seed Shattering Loci Provides Insights into Origin of Weedy Rice and Rice Domestication. Proc. J. Hered..

[47] Ji H., Kim S.R., Kim Y.H., Kim H., Eun M.Y., Jin I.D., Cha Y.S., Yun D.W., Ahn B.O., Lee M.C. (2010). Inactivation of the CTD Phosphatase-like Gene *OsCPL1* Enhances the Development of the Abscission Layer and Seed Shattering in Rice. Plant J..

[48] Lin Z., Griffith M.E., Li X., Zhu Z., Tan L., Fu Y., Zhang W., Wang X., Xie D., Sun C. (2007). Origin of Seed Shattering in Rice (*Oryza sativa* L.). Planta.

[49] Zhang Y.L., Xia Q.Y., Jiang X.Q., Hu W., Ye X.X., Huang Q.X., Yu S.B., Guo A.P., Lu B.R. (2022). Reducing Seed Shattering in Weedy Rice by Editing *SH4* and QSH1 Genes: Implications in Environmental Biosafety and Weed Control through Transgene Mitigation. Biology.

[50] Thurber C.S., Reagon M., Gross B.L., Olsen K.M., Jia Y., Caicedo A.L. (2010). Molecular Evolution of Shattering Loci in U.S. Weedy Rice. Mol. Ecol..

[51] Song B.K., Chuah T.S., Tam S.M., Olsen K.M. (2014). Malaysian Weedy Rice Shows Its True Stripes: Wild *Oryza* and Elite Rice Cultivars Shape Agricultural Weed Evolution in Southeast Asia. Mol. Ecol..

[52] Huang Z., Kelly S., Matsuo R., Li L.F., Li Y., Olsen K.M., Jia Y., Caicedo A.L. (2018). The Role of Standing Variation in the Evolution of Weedines Traits in South Asian Weedy Rice (*Oryza* spp.). G3 Genes Genomes Genet..

[53] Wedger M.J., Pusadee T., Wongtamee A., Olsen K.M. (2019). Discordant Patterns of Introgression Suggest Historical Gene Flow into Thai Weedy Rice from Domesticated and Wild Relatives. J. Hered..

[54] Komiya R., Yokoi S., Shimamoto K. (2009). A Gene Network for Long-Day Flowering Activates *RFT1* Encoding a Mobile Flowering Signal in Rice. Development.

[55] Hayama R., Yokoi S., Tamaki S., Yano M., Shimamoto K. (2003). Adaptation of Photoperiodic Control Pathways Produces Short-Day Flowering in Rice. Nature.

[56] Thurber C.S., Reagon M., Olsen K.M., Jia Y., Caicedo A.L. (2014). The Evolution of Flowering Strategies in Us Weedy Rice. Am. J. Bot..

[57] Shivrain V.K., Burgos N.R., Scott R.C., Gbur E.E., Estorninos L.E., McClelland M.R. (2010). Diversity of Weedy Red Rice (.) in Arkansas, U.S.A. in Relation to Weed Management. Crop Prot..

[58] Reagon M., Thurber C.S., Olsen K.M., Jia Y., Caicedo A.L. (2011). The Long and the Short of It: *SD1* Polymorphism and the Evolution of Growth Trait Divergence in U.S. Weedy Rice. Mol. Ecol..

[59] Gu X.Y., Foley M.E., Horvath D.P., Anderson J.V., Feng J., Zhang L., Mowry C.R., Ye H., Suttle J.C., Kadowaki K.I. (2011). Association between Seed Dormancy and Pericarp Color Is Controlled by a Pleiotropic Gene That Regulates Abscisic Acid and Flavonoid Synthesis in Weedy Red Rice. Genetics.

[60] Nagao S., Takahashi M.-E., Miyamoto T. (1957). Genetical studies on rice plant, xxi. biochemical studies on red rice pigmentation. Jpn. J. Genet..

[61] Furukawa T., Maekawa M., Oki T., Suda I., Iida S., Shimada H., Takamure I., Kadowaki K.I. (2007). The *Rc* and *Rd* Genes Are Involved in Proanthocyanidin Synthesis in Rice Pericarp. Plant J..

[62] Brooks S.A., Yan W., Jackson A.K., Deren C.W. (2008). A Natural Mutation in *Rc* Reverts White-Rice-Pericarp to Red and Results in a New, Dominant, Wild-Type Allele: *Rc-g*. Theor. Appl. Genet..

[63] Lee D., Lupotto E., Powell W. (2009). G-String Slippage Turns White Rice Red. Genome.

[64] Cui Y., Song B.K., Li L.F., Li Y.L., Huang Z., Caicedo A.L., Jia Y., Olsen K.M. (2016). Little White Lies: Pericarp Color Provides Insights into the Origins and Evolution of Southeast Asian Weedy Rice. G3 Genes Genomes Genet..

[65] Lu B.R., Li J., Lee D., Xu H., Zhang L., Dongchen W., Xiong H., Zhu Q., Zhang X., Chen L. (2014). Genetic Differentiation of Asian Weedy Rice Revealed with InDel Markers. Crop Sci..

[66] Li M.-B., Wang H., Cao L.-M. (2015). Evaluation of Population Structure, Genetic Diversity and Origin of Northeast Asia Weedy Rice Based on Simple Sequence Repeat Markers. Rice Sci..

[67] Cao Q., Lu B.R., Xia H., Rong J., Sala F., Spada A., Grassi F. (2006). Genetic Diversity and Origin of Weedy Rice (*Oryza sativa f. Spontanea*) Populations Found in North-Eastern China Revealed by Simple Sequence Repeat (SSR) Markers. Ann. Bot..

[68] Londo J.P., Schaal B.A. (2007). Origins and Population Genetics of Weedy Red Rice in the USA. Mol. Ecol..

[69] Grimm A., Fogliatto S., Nick P., Ferrero A., Vidotto F. (2013). Microsatellite Markers Reveal Multiple Origins for Italian Weedy Rice. Ecol. Evol..

[70] Teresa Federici M., Vaughan D., Tomooka N., Kaga A., Wang Wang X., Doi K., Francis M., Zorrilla G. (2001). Analysis of Uruguayan Weedy Rice Genetic Diversity Using AFLP Molecular Markers. Electron. J. Biotechnol..

[71] Li L.F., Li Y.L., Jia Y., Caicedo A.L., Olsen K.M. (2017). Signatures of Adaptation in the Weedy Rice Genome. Nat. Genet..

[72] He Q., Kim K.W., Park Y.J. (2017). Population Genomics Identifies the Origin and Signatures of Selection of Korean Weedy Rice. Plant Biotechnol. J..

[73] Jia Y., Jia M.H. (2021). Physiological, Ecological and Genetic Interactions of Rice with Harmful Microbes. Cereal Grains.

[74] Zhao H., Liu Y., Jia M.H., Jia Y. (2022). An Allelic Variant of the Broad-Spectrum Blast Resistance Gene *Ptr* in Weedy Rice Is Associated with Resistance to the Most Virulent Blast Race IB-33. Plant Dis..

[75] Zhao H., Wang X., Jia Y., Minkenberg B., Wheatley M., Fan J., Jia M.H., Famoso A., Edwards J.D., Wamishe Y. (2018). The Rice Blast Resistance Gene *Ptr* Encodes an Atypical Protein Required for Broad-Spectrum Disease Resistance. Nat. Commun..

[76] Liu Y., Qi X., Gealy D.R., Olsen K.M., Caicedo A.L., Jia Y. (2015). QTL Analysis for Resistance to Blast Disease in U.S. Weedy Rice. Mol. Plant-Microbe Interact..

[77] Goad D.M., Jia Y., Gibbons A., Liu Y., Gealy D., Caicedo A.L., Olsen K.M. (2020). Identification of Novel QTL Conferring Sheath Blight Resistance in Two Weedy Rice Mapping Populations. Rice.

[78] Jia L., Yan W., Zhu C., Agrama H.A., Jackson A., Yeater K., Li X., Huang B., Hu B., McClung A. (2012). Allelic Analysis of Sheath Blight Resistance with Association Mapping in Rice. PLoS ONE.

[79] Lee S., Jia Y., Jia M., Gealy D.R., Olsen K.M., Caicedo A.L. (2011). Molecular Evolution of the Rice Blast Resistance Gene *Pi*-*ta* in Invasive Weedy Rice in the USA. PLoS ONE.

[80] Jia Y., McAdams S.A., Bryan G.T., Hershey H.P., Valent B. (2000). Direct Interaction of Resistance Gene and Avirulence Gene Products Confers Rice Blast Resistance. EMBO J..

[81] Bryan G.T., Wu K.-S., Farrall L., Jia Y., Hershey H.P., Mcadams S.A., Faulk K.N., Donaldson G.K., Tarchini R., Valent B. (2000). A Single Amino Acid Difference Distinguishes Resistant and Susceptible Alleles of the Rice Blast Resistance Gene Pi-Ta Plant Cell 2000, 12, 2033–2045. e Pi-Ta Plant Cell.

[82] Rauf A., Imran M., Abu-Izneid T., Iahtisham-Ul-Haq, Patel S., Pan X., Naz S., Sanches Silva A., Saeed F., Rasul Suleria H.A. (2019). Proanthocyanidins: A Comprehensive Review. Biomed. Pharmacother..

[83] Grassi D., Necozione S., Lippi C., Croce G., Valeri L., Pasqualetti P., Desideri G., Blumberg J.B., Ferri C. (2005). Cocoa Reduces Blood Pressure and Insulin Resistance and Improves Endothelium-Dependent Vasodilation in Hypertensives. Hypertension.

[84] Heiss C., Kleinbongard P., Dejam A., Perré S., Schroeter H., Sies H., Kelm M. (2005). Acute Consumption of Flavanol-Rich Cocoa and the Reversal of Endothelial Dysfunction in Smokers. J. Am. Coll. Cardiol..

[85] Taubert D., Roesen R., Lehmann C., Jung N., Schö E. (2007). Effects of Low Habitual Cocoa Intake on Blood Pressure and Bioactive Nitric Oxide A Randomized Controlled Trial. Jama.

[86] Xu T.Y., Sun J., Chang H.L., Zheng H.L., Wang J.G., Liu H.L., Yang L.M., Zhao H.W., Zou D.T. (2017). QTL Mapping for Anthocyanin and Proanthocyanidin Content in Red Rice. Euphytica.

